# Effect of *Issatchenkia terricola* WJL-G4 on Deacidification Characteristics and Antioxidant Activities of Red Raspberry Wine Processing

**DOI:** 10.3390/jof8010017

**Published:** 2021-12-27

**Authors:** Hongying He, Yuchen Yan, Dan Dong, Yihong Bao, Ting Luo, Qihe Chen, Jinling Wang

**Affiliations:** 1 School of Forestry, Northeast Forestry University, No. 26, Hexing St., Harbin 150040, China; Nuyoah0820@163.com (H.H.); yanyuchen69619@nefu.edu.cn (Y.Y.); 957331735@nefu.edu.cn (D.D.); baoyihong@163.com (Y.B.); 2 Key Laboratory of Forest Food Resources Utilization of Heilongjiang Province, No. 26, Hexing St., Harbin 150040, China; 3 State Key Laboratory of Food Science and Technology, Nanchang University, No. 999, Xuefu St., Nanchang 330047, China; ting.luo@ncu.edu.cn; 4 Department of Food Science and Nutrition, Zhejiang University, Hangzhou 310058, China

**Keywords:** red raspberry wine, *Issatchenkia terricola* WJL-G4, deacidification, phenolic compounds, antioxidant activity

## Abstract

Our previous study isolated a novel *Issatchenkia terricola* WJL-G4, which exhibited a potent capability of reducing citric acid. In the current study, *I. terricol*a WJL-G4 was applied to decrease the content of citric acid in red raspberry juice, followed by the red raspberry wine preparation by *Saccharomyces cerevisiae* fermentation, aiming to investigate the influence of *I. terricola* WJL-G4 on the physicochemical properties, organic acids, phenolic compounds and antioxidant activities during red raspberry wine processing. The results showed that after being treated with *I. terricola* WJL-G4, the citric acid contents in red raspberry juice decreased from 19.14 ± 0.09 to 6.62 ± 0.14 g/L, which was further declined to 5.59 ± 0.22 g/L after *S. cerevisiae* fermentation. Parameters related to CIELab color space, including L*, a*, b*, h°, and ∆E* exhibited the highest levels in samples after *I. terricola* WJL-G4 fermentation. Compared to the red raspberry wine pretreated without deacidification (RJO-SC), wine pretreated by *I. terricola* WJL-G4 (RJIT-SC) exhibited significantly decreased contents of gallic acid, cryptochlorogenic acid, and arbutin, while significantly increased contents of caffeic acid, sinapic acid, raspberry ketone, quercitrin, quercetin, baicalein, and rutin. Furthermore, the antioxidant activities including DPPH· and ABTS^+^· radical scavenging were enhanced in RJIT-SC group as compared to RJO-SC. This work revealed that *I. terricola* WJL-G4 had a great potential in red raspberry wine fermentation.

## 1. Introduction

Red raspberry (*Rubus idaeus* L.) is known as an important commercial fruit with high contents of health-beneficial constituents and unique flavor [1]. It was reported that numerous bioactive components such as phenolics, flavones, anthocyanins, and vitamins are present in mature raspberry fruits, and these components contributed to the antioxidant capacities of these fruits [2]. Studies showed that red raspberry has positive effects on biological activities linked to reducing the risk for cancer, cardiovascular disease, diabetes mellitus and Alzheimer’s disease [3,4,5]. Polyphenol extracts from red raspberry whole fruit or pulp attenuated high-fat diet induced obesity in mice [6,7].

The red raspberry has been an emerging fruit crop in China over the last decades, although, for thousands of years, dried red raspberries produced in eastern China have been used for medicinal purposes. In the recent years, red raspberry has been widely cultivated in China, because of its unique flavor, taste, texture, and health benefits [8]. The production of red raspberries is steadily increasing [9]. However, industrial development of red raspberry products got restricted due to the limitation in preservation and processing techniques [10]. In the international market, the processed products of red raspberry include food drinks [11], baked products [12], leisure ready-to-eat products [13], frozen foods [14], while the research on red raspberry products in China is limited.

Red raspberry wine fermentation has been an alternative method to preserve nutrition and extend shelf life of red raspberry, not only effectively retaining the original flavor and active substances with antioxidant, hypoglycemia, prevention of cardiovascular diseases functions, but also enriching the market of processed red raspberry products. Red raspberry wine has the characteristics of pleasing sensory qualities, high nutritional value, and strong antioxidant function, which is in line with the green and healthy standards of modern people. However, high content of organic acid in red raspberry, mainly citric acid (22.87 g/L, accounting for 90.9% of the total acid) [15], caused excessive sour taste of red raspberry wine, and led to growth difficultly of microorganisms during fermentation. Therefore, to reduce the content of citric acid in the red raspberry wine has become an urgent problem.

The most common biological deacidification methods, including malo-lactic fermentation and malo-alcohol fermentation, can not apply to degrade citric acid [16,17]. Simultaneously, physical and chemical deacidification lead to poor quality, such as unstable wine body, bitter taste, loss of flavor substances, beneficial ingredients and color adsorption [18]. Therefore, biodegradation of citric acid has gradually become a new breakthrough in the process of winemaking. In our previous study, we isolated a novel characterized *I. terricol*a WJL-G4 from red raspberry fruits, which exhibited a potent capability of reducing the total acid contents from 25.16 g/L to 9.96 g/L within 8 d static fermentation of red raspberry juice [18,19]. However, the influence of *I*. *terricol*a WJL-G4 on red raspberry wine processing is not clarified. Other researches reported citric acid degradation strain JT-1-3, which was identified to be *Pichia fermentans* and showed strong ability to degrade citric acid [20] and improve kiwifruit wine quality. In our previous research, the fermentation of red raspberry juice with *I. terricola* WJL-G4 potently reduced citric acid concentration and increased the content of total flavonoid [15].

Based on the above research, we hypothesized that fermentation with *I. terricola* WJL-G4 could reduce citric acid content and improve the quality of red raspberry wine. In this work, the effects of deacidification using *I. terricola* WJL-G4 followed by *S. cerevisiae* wine fermentation on the physicochemical properties, organic acids, phenolic compounds and antioxidant activities of red raspberry wine were investigated in order to evaluate the use potential of *I. terricola* WJL-G4 on the characteristics of red raspberry wine.

## 2. Materials and Methods

### 2.1. Materials

Red raspberries of the cultivar “Autumn Bless”, one abundant raspberry species in Northeast China, were obtained from a local farm (Harbin, China) in August 2020. The fruits with similar maturity were hand-picked from different trees and then snap-frozen. Frozen red raspberries (40 kg) were mixed, transported to the laboratory in ice box and stored at −80 °C until use. All the standards for high performance liquid chromatography (HPLC) analysis, including rutin, quercetin, chlorogenic acid, ellagic acid, raspberry ketone, epicatechin, catechinic acid, syringic acid, gallic acid, caffeic acid, luteolin, citric acid, L-malic acid, succinic acid, α-ketoglutarate, and so on (HPLC grade), were obtained from Shanghai YuanYe Biotechnology (Shanghai, China).

### 2.2. Yeast Strains and Culture Media

*I. terricola* WJL-G4 was screened and isolated from fresh red raspberry (Chinese patent No. 2019113316700) after morphological and molecular biological identification and stored at China General Microbiological Culture Collection Center (CGMCC), No. 18712 [19]. *S. cerevisiae* BV818 (*S. cerevisiae* BV818) was purchased from Angel Yeast Co. Ltd. (Yichang, China). Wallerstein Laboratory (WL) citric acid medium (WLC) (1% citric acid, 0.5% yeast extract, 0.05% Mg_2_SO_4_) was used to cultivate *I. terricola* WJL-G4. Yeast extract peptone dextrose medium (YPD) (1% yeast extract, 2% yeast peptone, 2% glucose, *w*/*v*) was utilized to cultivate *S. cerevisiae* BV818.

### 2.3. Samples Preparation

To keep the consistency of sampling, at least 6 kg red raspberry fruits were milled after natural thawing. After an enzymatic treatment (pectinase, 40,000 U/g, enzyme:substrate = 0.02:100, *w*/*w*) at a temperature of 50 °C for 2 h, the obtained red raspberry pulp was incubated at 90 °C for 5 min for enzyme deactivation, followed by filtration using an 8-layer gauze and pasteurization at 80 °C for 15 min. Fermentation was performed in 250 mL glass bottles after yeasts were inoculated according to the designed protocols (next paragraph). Wine fermentation temperature was controlled between 24 °C and 26 °C (sample size was 3).

**Figure 1:**
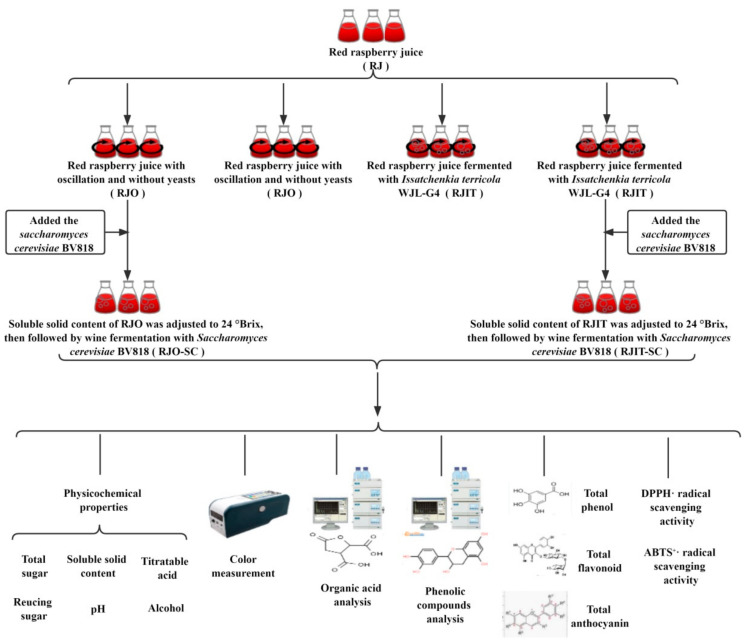
Experimental design of this work. RJ: red raspberry juice; RJO: red raspberry juice with oscillation (28 °C, 36 h, 240 r/min) and without yeasts; RJO-SC: soluble solid content of RJO was adjusted to 24 *°*Brix, then followed by wine fermentation with *S.cerevisiae* BV818 (24–26 °C, 15 d, 0.1% inoculation); RJIT: acid-reducing fermentation of RJ with *I. terricola* WJL-G4 (28 °C, 36 h, 240 r/min, 0.1% inoculation); and RJIT-SC: soluble solid content of RJIT was adjusted to 24 *°*Brix, then followed by wine fermentation with *S.cerevisiae* BV818 (24–26 °C, 15 d, 0.1% inoculation).

Subsequently, the red raspberry juice was equally divided into five different treatment samples (Figure 1): (i) RJ: red raspberry juice; (ii) RJO: red raspberry juice with oscillation (28 °C, 36 h, 240 r/min) and without yeasts; (iii) RJIT: acid-reducing fermentation of RJ with *I. terricola* WJL-G4 (28 °C, 36 h, 240 r/min, 0.1% inoculation); (iv) RJO-SC: soluble solid content of RJO was adjusted to 24 *°*Brix, then followed by wine fermentation with *S.cerevisiae* BV818 (24–26 °C, 15 d, 0.1% inoculation) and (v) RJIT-SC: soluble solid content of RJIT was adjusted to 24 *°*Brix, then followed by wine fermentation with *S.cerevisiae* BV818 (24–26 °C, 15 d, 0.1% inoculation). RJ, RJO, RJIT and RJO-SC were set as the control groups compared to RJIT-SC. After treatment, the supernatant was harvested with centrifugation at 4000 r/min for 15 min for further analysis.

### 2.4. Physicochemical Properties

Total sugar, reducing sugar, soluble solid content (SSC), alcoholic degree, pH value, and titratable acid were assayed applying the methods recommended by the International Organization of Vine and Wine [21]. Color measurements of samples were determined using the CIELab systems, as measured by the CM-5 Spectrophotometer color meter (Konica Minoita, Japan). L^*^ indicates lightness, where white = 100 and black = 0. L* (lightness), a* (from green to red), b* (from blue to yellow), C* (chroma or saturation-a vector from grey to saturated color) as square root of (a*^2^ + b*^2^), and h° (color shade) was calculated as arctan (b*/a*), ∆E* = [(∆L*)^2^ + (∆a*)^2^ + (∆b*)^2^]^1/2^ in CIELab unit [22].

### 2.5. Determination of Organic Acids

The organic acids in samples were analyzed by using Agilent 1260 Infinity II HPLC equipped with a DAD detector (Agilent Technologies Inc., Santa Clara, CA, USA). Separation and quantification of organic acids were performed at 210 nm with an Agilent Poroshell 120 EC-C18 column (4.6 × 150 mm, 4 μm). Two eluents, filtered through a 0.45 μm durapore membrane pore filter were used as mobile phases: eluent A: 0.5% (*w*/*v*) KH_2_PO_4_ (pH 2.3), eluent B: methanol, the elution condition was isostatic elution with A:B of 97:3 (*v*/*v*). Injection volume was 10 μL, flow rate was 0.7 mL/min, and column temperature was 35 °C.

### 2.6. Determination of the Total Phenol Contents

Total phenol contents of samples were determined using Folin-Ciocalteu method [23] with minor modifications. Briefly, samples were firstly tenfold diluted in distilled water (1:9, *v*/*v*). Diluted samples (1 mL) were mixed with 4 mL of sodium carbonate solution (7.5%, *w*/*v*) and 5 mL of 10% (*v*/*v*) Folin-Ciocalteu reagent using distilled water and was allowed to stand at room temperature for 1 h. The absorbance of mixtures was measured at 765 nm. A calibration curve was obtained using 0–100 μg gallic acid (GAE) /mL and was used to calculate the total phenol contents of samples. Total phenol contents in samples were calculated by regression equation and expressed as mg GAE/L.

### 2.7. Determination of the Total Flavonoid Contents

Total flavonoid contents of samples were evaluated using aluminium nitrate nonahydrate [24]. Rutin standard (28 mg, dried to a constant weight at 120 °C) was diluted with 70% (*v*/*v*) ethanol solution in a 50 mL volumetric flask. Then, 0.1, 0.2, 0.4, 0.6, 0.8 and 1.0 mL of rutin solutions were respectively mixed with 0.4 mL of 5% (*w*/*v*) sodium nitrite solution in 25 mL calibration test tubes, followed by the addition of 0.4 mL of 10% (*w*/*v*) aluminium nitrate solutions. After standing for 6 min, 4 mL aliquot of 1 M sodium hydroxide solution (NaOH) was further added. The mixtures were diluted to 25 mL with 70% (*v*/*v*) ethanol solution. Ten minutes later, the absorbance of rutin standard solutions was measured at 510 nm with reagent blank as reference and regression equation was calculated. To determine total flavonoid contents in samples, 1 mL of sample solution was put into a 25 mL calibration test tube and treated according to the above-mentioned operation. Total flavonoid contents in samples were calculated by regression equation and expressed as mg Rutin/L.

### 2.8. Determination of the Total Anthocyanin Contents

Total anthocyanin contents of samples were directly quantified by pH differential method [25]. Briefly, 1 mL samples were kept in constant volume to 10 mL with potassium chloride solution or sodium acetate solution (pH 1.0: 1.49 g KCl dissolved in 100 mL distilled water and adjusted to pH 1.0 with 1 M HCl, or sodium acetate buffer pH 4.5: 1.64 g CH_3_COONa·3H_2_O dissolved in 10 mL distilled water and adjusted to pH 4.5 with 1 M HCl). Then the absorbance was measured both at 520 and 700 nm after incubation for 20 min.

$$C(mg/L)=\frac{A\;\times \;M}{\varepsilon \;\times \;L}\;\times \;DF\;\times \;1000$$

*A* = absorption value, *M* = molecular weight (449 g/mol for cyd-3-glc), *ε* = molar extinction coefficient (26,900 L/mol cm for cyd-3-glc at pH 1.0), *DF* = dilution factor, and, *L* = path length of the cuvette (1 cm).

### 2.9. Identification of Phenolic Compounds by HPLC

The phenolic compounds confirmation analysis by HPLC (1260 Infinity ll, Agilent Technologies, Santa Clara, CA, USA) was accomplished by Agilent ZORBAX Eclipse Plus C18 column (250 × 4.6 mm, 5 μm). The phenolic compounds were separated under gradient conditions with a flow rate of 0.8 mL/min. Column temperature was 35 °C and volume injection was 10 µL. The wavelength was 280 nm. The solvents used in the elution process were an aqueous solution of 100% methanol (phase A) and 0.02% (*v*/*v*) formic acid (phase B). The samples were eluted according to the linear gradient, the phase-time program was as follows: 0–10% A (0–5 min), 10–20% A (5–10 min), 10–35% A (10–20 min), 35–40% (20–35 min), 40–75% A (35–40 min), 75–10% A (40–45 min). The peak areas of the samples were compared with those of the internal standards and quantified [26].

### 2.10. Determination of Antioxidant Activities

#### 2.10.1. DPPH Free Radical Scavenging Ability

DPPH· free radical scavenging abilities were performed using the method described by Ekumah [27] with slight modifications. DPPH· solution (1 mL, 0.025 g/L) was added to 0.5 mL sample, allowed to stand for 30 min in the dark at room temperature and the absorbance was recorded as sample group. Methyl alcohol (1 mL) was added to 0.5 mL sample and reacted for 30 min in the dark at room temperature and the absorbance was recorded as control group. Methyl alcohol (0.5 mL) and 1 mL DPPH· solution were mixed as the blank group. All absorbance was measured at a wavelength of 517 nm. The radical scavenging activity was calculated as follows:

(1) $$\;\mathrm{Radical}\;\mathrm{scavenging}\;\mathrm{activity}\;(\%)\;=\;(1-\frac{A_{sample}\;-\;A_{control}}{A_{\mathrm{blank}}})\;\times \;100\%$$

A*_sample_*: The absorbance of the sample group at 517 nm;

A*_control_*: The absorbance of the control group at 517 nm;

A*_blank_*: The absorbance of the blank group at 517 nm.

#### 2.10.2. ABTS^+^ Free Radical Scavenging Ability

ABTS^+^·free radical scavenging abilities of the samples were measured as previously described with slight modifications [28]. Briefly, 7 mM ABTS^+^·solution was mixed with 140 mM potassium persulfate aqueous solution to generate ABTS^+^·radical cation. The mixture was kept in the dark at room temperature for 12 h. Before the measurements, the resultant ABTS^+^·solution was diluted with ethanol to an absorbance of 0.70 ± 0.02 at 734 nm. Sample (0.5 mL) was mixed with 1 mL of the diluted ABTS^+^ solution and the absorbance was recorded as sample group. Sample (0.5 mL) was mixed with 1 mL of the ethanol and the absorbance was recorded as control group. Ethanol (0.5 mL) mixed with 1 mL of the diluted ABTS^+^· solution was used as blank group. Afterwards, the absorbance was measured at 734 nm. The radical scavenging activity represented the percentage of ABTS^+^·free radical inhibition and was calculated with Formula (1).

A*_sample_*: The absorbance of the sample group at 734 nm;

A*_control_*: The absorbance of the control group at 734 nm;

A*_blank_*: The absorbance of the blank group at 734 nm.

### 2.11. Statistical Analyses

Each experiment was repeated three times, and the results were expressed as the means ± standard deviation. SPSS 20.0 (V.26.0, SPSS Inc., Chicago, IL, USA) was used to analyze variance, and Duncan’s multiple comparison test was used to analyze the differences between the experimental groups. *p* < 0.05 was considered statistically significant.

## 3. Results and Discussion

### 3.1. Effect of I. terricola WJL-G4 on Physicochemical Properties of Red Raspberry Wines

The physicochemical properties of samples with different treatments (RJ, RJO, RJIT, RJO-SC, RJIT-SC) showed significant differences (Table 1). The contents of total sugar and reducing sugar declined in RJIT, RJO-SC and RJIT-SC, because energy from carbohydrate metabolism is crucially important for yeasts growth and reproduction during fermentation [29]. The decline in SSC and total sugar coincided with the great consumption of sugar by *I*. *terricola* WJL-G4 and *S.* *cerevisiae* BV818. The alcoholic degree of red raspberry wines, including RJO-SC and RJIT-SC, reached 9.93 ± 0.47 and 10.23 ± 0.87 (%, *v*/*v*), respectively. The results indicated that *I. terricola* WJL-G4 produced a limited amount of ethanol, which was only 3.90 ± 0.12 (%, *v*/*v*) in RJIT. Because of the inherent properties of non-*Saccharomyces cerevisiae*, its alcohol-producing capacity was generally lower than that of *S. cerevisiae* [30]. The titratable acid content of RJO-SC was 19.40 ± 0.15 g/L, slightly lower than that of RJO (22.86 ± 0.21 g/L), while significantly higher than that of RJIT and RJIT-SC with mean values of 11.83 ± 0.97 and 9.22 ± 0.17 g/L, respectively. On the other hand, RJIT and RJIT-SC showed significantly higher pH values in comparison with RJO and RJO-SC. These results indicated that *I. terricola* WJL-G4 fermentation reduced titratable acid and increased pH values of red raspberry wines.

Color is also a basic quality of fruit drinks. ΔE* reflects the relation of total color difference among L*, a* and b* [31]. The values of CIELab in different samples were reported in Table 1. Among samples, RJIT and RJIT-SC had significantly higher values of L*, a*, b*, h°, and ΔE*, which was 24.76 ± 0.93, 56.55 ± 0.87, 42.17 ± 1.74, 36.76 ± 0.71, and 17.86 ± 0.15 in RJIT and 24.95 ± 0.99, 53.66 ± 0.81,41.32 ± 1.53, 37.39 ± 0.96, and 15.93 ± 1.99 in RJIT-SC, respectively, compared to other samples, indicating that *I. terricola* WJL-G4 fermentation significantly promoted the color of fermented samples. Non-*Saccharomyces cerevisiae* has been reported to play a positive role in wine color. Morata and Benito reported that fermentation with non-*Saccharomyces cerevisiae* strains with high activity of ethyl p-hydroxycinnamate decar-decarboxylase (HCDC) could increase the formation of styrene-pyranoanthocyanins in wine fermentation [32]. Sequential fermentation with *Pichia guilliermondii* and *S. cerevisiae* was used to enhance the production of styrene-pyranoanthocyanins, thus making the color of wine more stable [33].

### 3.2. Effect of I. terricola WJL-G4 on Organic Acids Contents

Organic acids have been reported to be important factors influencing aroma, taste and microbiological stability in wine [34]. The contents of 7 organic acids in samples were presented in Table 2. Except for succinic acid, other organic acids in the wines (RJO-SC and RJIT-SC) showed a downward trend compared to RJ. Compared to RJO-SC, the concentrations of oxalic acid, tartaric acid, α-ketoglutaric acid in RJIT-SC were decre- ased from 0.57 ± 0.03 to 0.45 ± 0.01 g/L, 0.49 ± 0.00 to 0.16 ± 0.00 g/L, and 0.28 ± 0.13 to 0.08 ± 0.00 g/L, respectively; especially, the contents of citric acid were reduced from 17.14 ± 0.16 to 5.59 ± 0.22 g/L. In addition, compared with RJ (0.69 ± 0.05 g/L), succinic acid concentrations in RJIT and RJIT-SC were increased to 1.74 ± 0.17 and 1.42 ± 0.22 g/L, respectively. During aging, succinic acid will combine with other substances to form ethyl succinic acid, which is beneficial to the aroma of wine, accentuates wine’s flavor and vinous character [35]. The contents of organic acids in RJO and RJIT showed a similar trend compared to that in RJO-SC and RJIT-SC, indicating that *I. terricola* WJL-G4 had the ability to metabolize organic acids, especially citric acid, when there was glucose or other absorbable carbon sources, therefore, it could be concluded that *I. terricola* WJL-G4 reduced organic acids significantly in red raspberry wine, yet *S. cerevisiae* BV818 had limited capacity to transform organic acids. Compared to our previous study, which showed that static fermentation with *I. terricola* WJL-G4 could degrade citric acid from 22.87 to 9.25 g/L in red raspberry juice after 8 days [15], *I. terricola* WJL-G4 showed more efficient potential to reduce citric acid during wine fermentation with a shorter time period and better acid-degradation ability with oscillation. The non-*Saccharomyce* yeast *Pichia fermentans* JT-1-3 was reported to have the ability to degrade citric acid from 12.3 to 11.0 g/L in kiwifruit wine, and to degrade citric acid by 43.35% in blueberry wine [20,36]. The reduction of organic acids may reduce the formation of “fermentation bouquet”, leading to a better wine taste, quality and purchase desire [37].

### 3.3. Effect of I. terricola WJL-G4 on Total Phenol, Flavonoid and Total Anthocyanin Contents of Red Raspberry Wines

**Figure 2:**
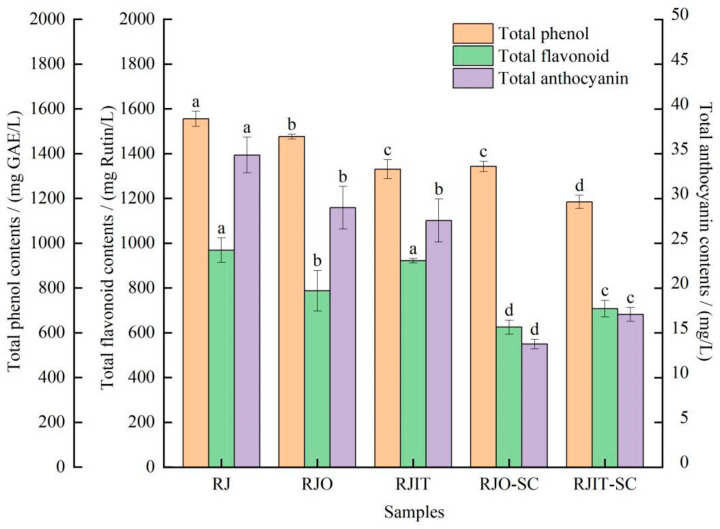
Effect of *I. terricola* WJL-G4 on total phenol, total flavonoid and total anthocyanin contents of red raspberry wines. Values were given as the means ± standard deviation (*n* = 3), and the different letters within the same color were significantly different (*p* < 0.05).

Polyphenols is a critical parameter of wine quality, which are not only related to the color, astringency, bitterness and other flavor quality parameters of wine, but also have good biological and antioxidant properties [38]. As shown in Figure 2, the initial content of total phenol in the RJ sample was the highest of 1555.10 ± 29.47 mg GAE/L. By the end of different processing, the total phenol contents in RJO, RJIT, RJO-SC and RJIT-SC were 1476.40 ± 8.58, 1330.55 ± 37.06, 1342.72 ± 19.11 and 1184.65 ± 26.12 mg GAE/L, respectively. The results indicated that both *I*. *terricola* WJL-G4 and *S. cerevisiae* could reduce the total phenol contents in red raspberry. Shu et al. [39] found that a detectable drop of almost 20% in phenolic contents after wine fermentation was probably due to the adsorption of phenols onto yeast cell walls and the reaction with cell wall proteins [40]. The decrease of phenolic acids in raspberry juice during fermentation might be due to microbial decomposition of macromolecular phenolic acids into small molecules or adherence of phenolic acids to yeast cell walls [41,42]. The highest amounts of total flavonoid were found in RJ and RJIT (Figure 2), consistent with our previous results [19]. Interestingly, RJIT-SC retained more total flavonoid content than RJO-SC. It could be concluded that the total flavonoid content in RJIT-SC was increased by *I. terricola* WJL-G4 fermentation.

Total anthocyanin contents in RJO and RJIT decreased from 34.86 ± 1.72 to 28.98 ± 1.94 and 27.54 ± 2.74 mg/L, respectively, compared to RJ, and showed no significant difference, as shown in Figure 2. Interestingly, total anthocyanin content in RJIT-SC was significantly higher than that in RIO-SC, although both were significantly decreased after wine fermentation. Anthocyanins have a variety of biological activities, including antioxidant, anti-inflammatory, anti-cardiovascular disease, anti-skin damage and protection of the reproductive system [43]. However, anthocyanins are sensitive to light, pH, temperature and other factors during fermentation and aging, resulting in poor preservation of sensory properties of wine and its antioxidative effects [25]. It was found that after 15 d and 30 d of storage, anthocyanin contents decreased by 66.4% and 90.0%, respectively [44]. The degradation of anthocyanin and polymerization between anthocyanin and other constituents (e.g., protein) might lead to the loss of total anthocyanin contents in raspberry juice during storage [45,46]. Yang et al. [47] found that fermentation significantly reduced the total contents of anthocyanin from 422.1 mg/L in strawberry juice to 235.5 mg/L in fermentation beverage. The loss of total anthocyanin during fermentation can be influenced by multiple factors. Anthocyanin may interact with other flavonoids, forming more stable pyranoanthocyanins, and the decrease in polarity of these compounds was accompanied by a decrease in solubility [48]. Therefore, a conclusion might be drawn that *I. terricola* WJL-G4 exhibited protective effects on total anthocyanin contents during wine fermentation.

### 3.4. Effect of I. terricola WJL-G4 on Phenolic Compounds of Red Raspberry Wines

Eighteen phenolic compounds, including 10 phenolic acids and 8 flavonoids, were identified and quantified by HPLC, as shown in Table 3. Of the compounds tested, only quercitrin was not identified in RJ and RJO. The phenolic profile varied between samples. Most phenolic compound contents were found decreased after fermentation in RJIT, RJO-SC and RJIR-SC, especially in RJO-SC and RJIT-SC. However, contents of caffeic acid, quercitrin, quercetin and raspberry ketone increased significantly after *I*. *terricola* WJL-G4 fermentation in both RJIT and RJIT-SC, compared to RJ, RJO and RJO-SC. Ellagic acid contents showed a slight decrease after wine fermentation. Compared to RJO-SC, contents of gallic acid, cryptochlorogenic acid and arbutin decreased significantly, yet caffeic acid, sinapic acid, rutin, quercitrin, quercetin, baicalein and raspberry ketone increased significantly in RJIT-SC.

Raspberry ketone, as the secondary metabolite of raspberry, was significantly increased in RJIT and RJIT-SC. Raspberry ketone contents in RJ, RJO, RJIT, RJO-SC and RJIT-SC were 5.48 ± 0.08, 5.45 ± 0.03, 20.19 ± 0.67, 3.76 ± 0.60 and 12.71 ± 1.27 mg/L, respectively. It might be concluded that fermentation with *I. terricola* WJL-G4 significantly increased the contents of raspberry ketone. Raspberry ketone is an important characteristic aroma component in ripe raspberries [49]. In addition, raspberry ketone has been used in the pharmaceutical makeup industry because of its skin-whiting property [50].

### 3.5. Effect of I. terricola WJL-G4 on DPPH and ABTS^+^·Radical Scavenging Activities of RED Raspberry Wines

Phenolic compounds exhibit different biological activities, especially antioxidant activities. They protect biomolecules from oxidative damage through free radical mediated reactions and inhibit oxidative chain reactions in a variety of ways, including direct quenching of reactive oxygen species, inhibition of enzymes and chelating metal ions [51]. Herein, different in vitro antioxidant activities were tested and shown in Table 4. A concentration effect relationship was found in all samples between concentrations and antioxidant activities. In particular, RJ showed the highest DPPH· and ABTS^+^· radical scavenging activities with IC50 values of 1.51 ± 0.04 and 13.25 ± 0.14 mg/mL, respectively, followed by RJIT with IC50 value of 1.84 ± 0.07 and 14.49 ± 0.24 mg/mL, respectively. The DPPH· and ABTS^+^·radical scavenging IC50 values in RJIT-SC were 2.10 ± 0.06 and 16.38 ± 0.13 mg/mL, compared to that of RJO-SC with 2.33 ± 0.06 and 17.26 ± 0.17 mg/mL. We might draw a conclusion that the fermentation treatment by *I. terricola* WJL-G4 could enhance the antioxidant activities of fermented red raspberry wine.

Su and Wang [20] found that during juice storage, the changes of antioxidant activity was likely to be associated with the degradation pathways of anthocyanin. Reports [52] showed that the total phenol and total flavonoid contents of wines exhibited the strongest correlations with antioxidant properties but that total anthocyanin contents exhibited weaker correlations. He et al. [53] found that hawthorn wine showed positive correlations between antioxidant activities and total anthocyanin, total phenol and total flavonoid contents, and the strongest correlation was observed between antioxidant activity and total phenol content.

**Figure 3:**
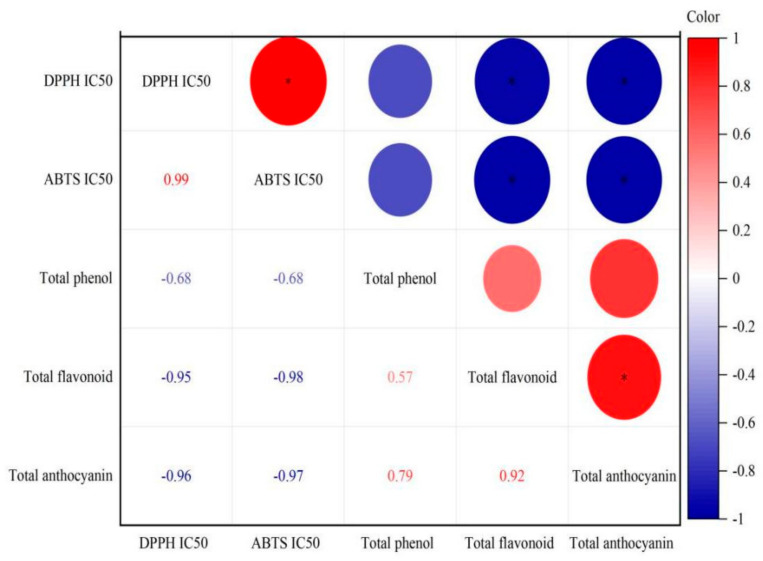
Correlation between total phenol, total flavonoid, total anthocyanin contents and antioxidant activities. The * indicated significantly different (*p* < 0.05). The number represented the Pearson correlation coefficient.

The present data were basically consistent with some of the results mentioned above, as shown in Figure 3. There was a good correlation between the antioxidant activities and the bioactive components of samples. The IC50 of DPPH· and ABTS^+^ free radical scavenging activities had significantly negative correlation with the contents of total flavonoid and total anthocyanin, with the correlation coefficients of −0.95, −0.96 and −0.98, −0.97 (*p* < 0.05), respectively; and they were negatively correlated with total phenol content with the correlation coefficients of −0.68 (*p* > 0.05). Total phenol had positive correlation with total flavonoid and total anthocyanin with the correlation coefficients of 0.57 and 0.79 (*p* > 0.05), respectively, yet total flavonoid had significantly positive correlation with total anthocyanin (correlation coefficient of 0.92, *p* < 0.05). This result confirmed with the previous conclusion that the higher the contents of total phenol, total flavonoid and total anthocyanin, the higher the antioxidant activity will be observed. The results showed that the contents of these active substances were important factors affecting the antioxidant capacity of red raspberry wine.

## 4. Conclusions

In this work, the fermentation with *I. terricola* WJL-G4 could significantly degrade citric acid, improve the color of the wine, enhance contents of total flavonoid and total anthocyanin, thus enhanced the antioxidant activities of red raspberry wine. Red raspberry wine produced with *I. terricola* WJL-G4 might have an improved taste due to citric acid reduction. In conclusion, *I. terricola* WJL-G4 showed great potential to be applied in red raspberry or other fruit wine production with high levels of citric acid. Further work may focus on the effect of *I. terricola* WJL-G4 fermentation on the aroma compounds of red raspberry wine during the wine processing.

## Figures and Tables

**Table 1:** Effect of *I. terricola* WJL-G4 on physicochemical properties of red raspberry wines.

	RJ	RJO	RJIT	RJO-SC	RJIT-SC
Total sugar/(g·L−1)	61.79 ± 5.33 ^a^	55.56 ± 1.29 ^b^	6.19 ± 0.41 ^c^	6.89 ± 0.74 ^c^	6.43 ± 0.43 ^c^
Reducing sugar/(g·L−1)	42.67 ± 3.06 ^a^	39.64 ± 1.76 ^b^	3.68 ± 0.24 ^c^	4.28 ± 0.23 ^c^	4.00 ± 0.46 ^c^
Soluble solid content/(*°*Brix)	9.15 ± 0.60 ^a^	8.00 ± 0.00 ^b^	3.00 ± 0.00 ^e^	5.63 ± 0.75 ^c^	4.75 ± 0.42 ^d^
Alcohol/(%, *v*/*v*)	n.d.	n.d.	3.90 ± 0.12 ^c^	9.93 ± 0.47 ^b^	10.23 ± 0.87 ^a^
Titratable acid/(g·L^−1^)	23.56 ± 1.70 ^a^	22.86 ± 0.21 ^ab^	11.83 ± 0.97 ^c^	19.40 ± 0.15 ^b^	9.22 ± 0.17 ^d^
pH	2.86 ± 0.01 ^e^	2.88 ± 0.01 ^d^	3.54 ± 0.12 ^b^	3.02 ± 0.03 ^c^	3.86 ± 0.10 ^a^
L*	16.99 ± 0.16 ^c^	19.7 ± 0.02 ^b^	24.76 ± 0.93 ^a^	20.04 ± 1.17 ^b^	24.95 ± 0.99 ^a^
a*	47.50 ± 0.01 ^d^	50.95 ± 0.01 ^c^	56.55 ± 0.87 ^a^	50.74 ± 1.56 ^c^	53.66 ± 0.81 ^b^
b*	28.97 ± 0.01 ^c^	33.62 ± 0.16 ^b^	42.17 ± 1.74 ^a^	34.26 ± 1.89 ^b^	41.32 ± 1.53 ^a^
C*	55.63 ± 0.01 ^c^	68.86 ± 0.02 ^a^	70.61 ± 1.75 ^a^	61.23 ± 2.35 ^b^	67.73 ± 1.57 ^a^
h°	31.37 ± 0.01 ^c^	33.41 ± 0.12 ^b^	36.76 ± 0.71 ^a^	34.01 ± 0.64 ^b^	37.39 ± 0.96 ^a^
ΔE*	n.a.	6.39 ± 0.13 ^b^	17.86 ± 0.15 ^a^	6.91 ± 2.69 ^b^	15.93 ± 1.99 ^a^

Values were given as the means ± standard deviation (*n* = 3), and the different letters within each row were significantly different (*p* < 0.05). CIE Lab coordinates: L* (lightness), a* (from green to red), b* (from blue to yellow), C* (chroma or saturation), and h*°* (hue angle). ΔE* = [(ΔL*)^2^ + (Δa*)^2^ + (Δb*)^2^]^1/2^ in CIELab unit. n.d.: Not detected. n.a.: Not applicable.

**Table 2:** Effect of *I. terricola* WJL-G4 on organic acids contents of red raspberry wines.

	RJ	RJO	RJIT	RJO-SC	RJIT-SC
Citric acid/(g·L^−1^)	19.14 ± 0.09 ^a^	18.80 ± 0.40 ^a^	6.62 ± 0.14 ^c^	17.14 ± 0.16 ^b^	5.59 ± 0.22 ^d^
Malic acid/(g·L^−1^)	1.25 ± 0.03 ^a^	1.10 ± 0.22 ^ab^	0.39 ± 0.04 ^c^	0.57 ± 0.18 ^c^	0.94 ± 0.08 ^b^
Oxalic acid/(g·L^−1^)	0.92 ± 0.01 ^a^	0.92 ± 0.07 ^a^	0.47 ± 0.01 ^c^	0.57 ± 0.03 ^b^	0.45 ± 0.01 ^c^
Tartaric acid/(g·L^−1^)	0.56 ± 0.01 ^b^	0.79 ± 0.06 ^a^	0.39 ± 0.03 ^c^	0.49 ± 0.00 ^b^	0.16 ± 0.00 ^d^
Succinic acid/(g·L^−1^)	0.69 ± 0.05 ^c^	0.81 ± 0.02 ^c^	1.74 ± 0.17 ^a^	0.09 ± 0.05 ^d^	1.42 ± 0.22 ^b^
α-Ketoglutaric acid/(g·L^−1^)	0.31 ± 0.12 ^a^	0.22 ± 0.15 ^a^b	0.16 ± 0.00 ^b^c	0.28 ± 0.13 ^ab^	0.08 ± 0.00 ^c^
Fumaric acid/(g·L^−1^)	0.02 ± 0.00 ^b^	0.03 ± 0.00 ^a^	n.d.	n.d.	n.d.

Values were given as the means ± standard deviation (*n* = 3), and the different letters within each row were significantly different (*p* < 0.05). n.d.: Not detected.

**Table 3:** Effect of *I*. *terricola* WJL-G4 on phenolic compounds of red raspberry wines.

Category	Contents/(mg·L^−1^)				
	RJ	RJO	RJIT	RJO-SC	RJIT-SC
Gallic acid	9.99 ± 0.30 ^a^	9.41 ± 0.12 ^a^	7.53 ± 0.23 ^b^	7.47 ± 0.51 ^b^	6.40 ± 1.12 ^c^
Cryptochlorogenic acid	90.92 ± 9.71 ^a^	95.17 ± 3.98 ^a^	54.45 ± 7.86 ^b^	88.56 ± 7.34 ^a^	46.56 ± 6.77 ^b^
p-Hydroxybenzonic acid	73.73 ± 8.12 ^a^	59.90 ± 5.44 ^b^	45.40 ± 4.03 ^c^	42.89 ± 1.57 ^c^	38.89 ± 3.02 ^c^
Chlorogenic acid	107.58 ± 4.10 ^a^	92.06 ± 2.25 ^b^	75.51 ± 9.99 ^c^	45.86 ± 1.06 ^d^	49.06 ± 5.11 ^d^
Neochlorogenic acid	311.23 ± 44.71 ^a^	260.18 ±20.93 ^b^	164.34 ± 7.95 ^c^	57.80 ± 7.42 ^d^	32.69 ± 5.70 ^d^
Caffeic acid	14.18 ± 0.13 ^cd^	14.79 ± 0.74 ^c^	21.21 ± 0.61 ^a^	13.38 ± 0.11 ^d^	16.43 ± 0.90 ^b^
Syringate	1.90 ± 0.05 ^ab^	1.98 ± 0.06 ^a^	1.74 ± 0.02 ^c^	1.88 ± 0.04 ^b^	1.83 ± 0.09 ^bc^
p-Coumaric acid	2.33 ± 0.11 ^b^	4.27 ± 0.35 ^a^	4.48 ± 0.31 ^a^	1.46 ± 0.13 ^c^	1.48 ± 0.12 ^c^
Ellagic acid	30.45 ± 0.12 ^a^	30.49 ± 0.11 ^a^	30.36 ± 0.08 ^a^	30.04 ± 0.18 ^b^	29.99 ± 0.14 ^b^
Sinapic acid	88.73 ± 4.90 ^a^	83.46 ± 1.47 ^a^	72.74 ± 5.49 ^b^	29.71 ± 5.28 ^d^	54.11 ± 9.49 ^c^
Arbutin	99.93 ± 7.70 ^a^	94.28 ± 5.03 ^a^	72.45 ± 2.14 ^c^	85.50 ± 5.37 ^b^	66.93 ± 5.18 ^c^
Rutin	11.29 ± 0.53 ^a^	11.83 ± 0.57 ^a^	12.13 ± 0.78 ^a^	2.48 ± 0.43 ^c^	3.45 ± 0.38 ^b^
Quercitrin	n.d.	n.d.	4.69 ± 0.22 ^a^	3.05 ± 0.30 ^c^	3.67 ± 0.20 ^b^
Quercetin	3.66 ± 0.11 ^bc^	3.98 ± 0.21 ^b^	4.75 ± 0.87 ^a^	2.99 ± 0.44 ^c^	4.52 ± 0.41 ^ab^
Luteolin	0.88 ± 0.06 ^b^	0.90 ± 0.03 ^b^	0.76 ± 0.05 ^b^	1.49 ± 0.32 ^a^	1.53 ± 0.26 ^a^
Kaempferol	-	-	-	-	-
Baicalein	3.95 ± 0.18 ^c^	4.13 ± 0.23 ^a^	3.64 ± 0.06 ^b^	3.13 ± 0.11 ^c^	3.71 ± 0.35 ^b^
Raspberry ketone	5.48 ± 0.08 ^c^	5.45 ± 0.03 ^c^	20.19 ± 0.67 ^a^	3.76 ± 0.60 ^d^	12.71 ± 1.27 ^b^

Values were given as the means ± standard deviation (*n* = 3), and the different letters within each row were significantly different (*p* < 0.05). n.d.: Not detected. -: the content’ was too low to calculate.

**Table 4:** Effect of *I*. *terricola* WJL-G4 on DPPH· and ABTS^+^· radical scavenging activities of red raspberry wines.

	RJ	RJO	RJIT	RJO-SC	RJIT-SC	VC
IC50 for DPPH/(mg/mL)	1.51 ± 0.04 ^d^	1.90 ± 0.24 ^bc^	1.84 ± 0.07 ^c^	2.33 ± 0.06 ^a^	2.10 ± 0.06 ^bc^	0.016 ± 0.01 ^f^
IC50 for ABTS^+^/(mg/mL)	13.25 ± 0.14 ^e^	15.16 ± 0.16 ^c^	14.49 ± 0.24 ^d^	17.26 ± 0.17 ^a^	16.38 ± 0.13 ^b^	0.028 ± 0.01 ^f^

Values were given as the means ± standard deviation (*n* = 3), and the different letters within each row were significantly different (*p* < 0.05). VC was Vitamin C (ascorbic acid).

## Data Availability

Not applicable.
